# 1324. Penicillin-Susceptibility among *Staphylococcus aureus* Skin-and-Soft-Tissue Infections at a Children’s Hospital

**DOI:** 10.1093/ofid/ofac492.1154

**Published:** 2022-12-15

**Authors:** C McNeil Jonathon, Lauren M Sommer, Marritta Joseph, Kristina G Hulten, Sheldon L Kaplan

**Affiliations:** Baylor College of Medicine, Houston, Texas; Baylor College of Medicine, Houston, Texas; Baylor College of Medicine, Houston, Texas; Baylor College of Medicine, Houston, Texas; Baylor College of Medicine, Houston, Texas

## Abstract

**Background:**

Shortly after its introduction into clinical practice, *Staphylococcus aureus* isolates gained resistance to penicillin via the acquisition of β-lactamases. By the 1960s, these strains became the dominant staphylococci in clinical practice. However, a number of centers have recently described an increase in the proportion of methicillin susceptible *S. aureus* (MSSA) which are also susceptible to penicillin (PSSA). In our center we have recently observed the emergence of PSSA in osteoarticular infections. Little data are available regarding the prevalence or impact of PSSA in pediatric skin-and-soft-tissue infections (SSTI).

**Methods:**

MSSA SSTI isolates were obtained through an ongoing surveillance study at Texas Children’s (TCH) from 1/2017-12/2021. Twenty community-acquired MSSA SSTI isolates were chosen at random from every six-month interval during the study period, for a total of 200 isolates to be screened. All isolates underwent PCR for *blaZ* β-lactamase, PVL genes and *agr* group. All *blaZ* negative isolates then underwent penicillin susceptibility testing using macrobroth dilution. Isolates which were *blaZ* negative and had a penicillin MIC ≤ 0.125 μg/ml were regarded as PSSA with the remainder regarded as penicillin-resistant MSSA (PR-MSSA).

**Results:**

During the study period 1701 MSSA SSTI isolates were collected with 200 examined for penicillin susceptibility. The overall median age of subjects was 4.1 years (IQR: 1.6-10.4). PSSA accounted for 9% of isolates during the study period; the annual frequency of PSSA varied from 5-17.5%. PSSA and PR-MSSA cases were similar with respect to age, demographics, anatomic site of infection and rates of prior antibiotic exposure. Subjects with SSTI secondary to PSSA more often had a prior history of SSTI, a diagnosis of abscess and were more often admitted to the hospital (**Figure**). Among PSSA, 94.4% belonged to *agr*I and 33.3% were PVL positive.

Penicillin-Susceptible S. aureus (PSSA) vs. Penicillin-Resistant Methicillin-Susceptible S. aureus (PR-MSSA) Pediatric SSTI Isolates

**Figure:**
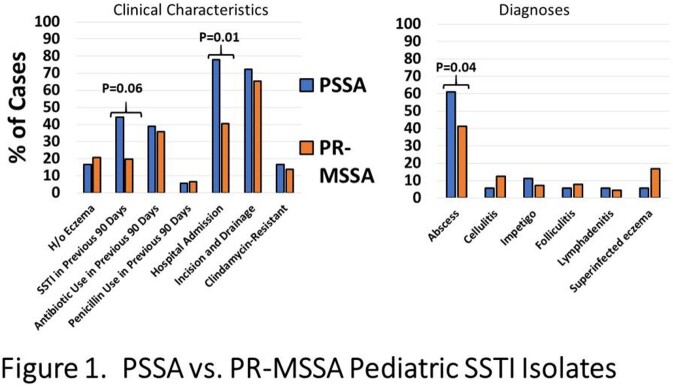

Comparison of the clinical features of PSSA and PR-MSSA SSTI isolates. PSSA were significantly associated with the diagnosis of abscess as well as hospital admission.

**Conclusion:**

PSSA account for a small but significant proportion of MSSA SSTI in children. Clinically distinguishing patients with PSSA and PR-MSSA is challenging. However, PSSA SSTI were associated with a greater rate of abscess formation and hospital admission, suggesting a high clinical impact of these infections. Further studies are needed to understand the optimal management of these infections.

**Disclosures:**

**Jonathon C. McNeil, MD**, Agency for Healthcare Research and Quality: Grant/Research Support|Allergan: Provided reagents for unrelated research|Nabriva: Site investigator for multicenter clinical trial **Kristina G. Hulten, PhD**, Me-med: Grant/Research Support|Pfizer: Grant/Research Support **Sheldon L. Kaplan, MD**, MeMed: Grant/Research Support|Pfizer: Grant/Research Support.

